# Perioperative goal-directed therapy and postoperative outcomes in patients undergoing high-risk abdominal surgery: a historical-prospective, comparative effectiveness study

**DOI:** 10.1186/s13054-015-0945-2

**Published:** 2015-06-19

**Authors:** Maxime Cannesson, Davinder Ramsingh, Joseph Rinehart, Aram Demirjian, Trung Vu, Shermeen Vakharia, David Imagawa, Zhaoxia Yu, Sheldon Greenfield, Zeev Kain

**Affiliations:** Department of Anesthesiology & Perioperative Care, University of California, Irvine, CA USA; Health Policy Research Institute, University of California, Irvine, CA USA; Department of Surgery, University of California, Irvine, CA USA; Department of Statistics, University of California, Irvine, CA USA

## Abstract

**Introduction:**

Perioperative goal-directed therapy (PGDT) may improve postoperative outcome in high-risk surgery patients but its adoption has been slow. In 2012, we initiated a performance improvement (PI) project focusing on the implementation of PGDT during high-risk abdominal surgeries. The objective of the present study was to evaluate the effectiveness of this intervention.

**Methods:**

This is a historical prospective quality improvement study. The goal of this initiative was to standardize the way fluid management and hemodynamic optimization are conducted during high-risk abdominal surgery in the Departments of Anesthesiology and Surgery at the University of California Irvine. For fluid management, the protocol consisted in standardized baseline crystalloid administration of 3 ml/kg/hour and any additional boluses based on PGDT. The impact of the intervention was assessed on the length of stay in the hospital (LOS) and post-operative complications (NSQIP database).

**Results:**

In the 1 year pre- and post-implementation periods, 128 and 202 patients were included. The average volume of fluid administered during the case was 9.9 (7.1–13.0) ml/kg/hour in the pre-implementation period and 6.6 (4.7–9.5) ml/kg/hour in the post-implementation period (*p* < 0.01). LOS decreased from 10 (6–16) days to 7 (5–11) days (*p* = 0.0001). Based on the multiple linear regression analysis, the estimated coefficient for intervention was 0.203 (SE = 0.054, *p* = 0.0002) indicating that, with the other conditions being held the same, introducing intervention reduced LOS by 18 % (95 % confidence interval 9–27 %). The incidence of NSQIP complications decreased from 39 % to 25 % (*p* = 0.04).

**Conclusion:**

These results suggest that the implementation of a PI program focusing on the implementation of PGDT can transform fluid administration patterns and improve postoperative outcome in patients undergoing high-risk abdominal surgeries.

**Trial registration:**

Clinicaltrials.gov NCT02057653. Registered 17 December 2013.

**Electronic supplementary material:**

The online version of this article (doi:10.1186/s13054-015-0945-2) contains supplementary material, which is available to authorized users.

## Introduction

Perioperative goal-directed therapy (PGDT) strategies based on cardiac output and/or oxygen delivery optimization have been shown to improve postoperative outcome in patients undergoing high-risk surgery [1–6]. This has been reported in both single center trials [5, 7], and quality improvement-based studies [3], and confirmed in published meta-analyses [2, 8]. Evidence supporting PGDT has been considered strong enough for this clinical practice to now be included in recommendations released by National Health Services in the UK [9, 10], by the French Society of Anesthesiology (Société Francaise d’Anesthésie Réanimation) [11], and by the European Society of Anaesthesiology [12]. In the United States, the Perioperative Surgical Home initiative [13, 14] also emphasizes the importance of “precise fluid management” during surgery. However, despite these recommendations, adoption of this practice has been slow, and to date few clinicians and institutions apply this concept for the management of high-risk surgery patients [15]. Consequently, the effectiveness of this approach in real life settings is not clearly understood.

The initiative described in this manuscript aimed to standardize the way fluid management and hemodynamic optimization are conducted during high-risk abdominal and pelvic surgeries, as a focus group that consisted of surgeons and anesthesiologists indicated much variability in this practice. The overall goal of the initiative was to study the effectiveness of a systematic implementation of PGDT strategies on postoperative length of stay (LOS) and on the incidence of postoperative complications following high-risk abdominal surgery. This current report presents historical-prospective, comparative effectiveness data on the effect of the PGDT up to 15 months after its implementation.

The initiative was supported by a grant from the Center for Health and Quality Innovation at the Office of the President of the University of California (Principal Investigator, Maxime Cannesson).

## Methods

This study was approved by the Institutional Review Board of the University of California Irvine Medical Center (IRB B: IRB@research.uci.edu; HS# 2011–8140, “Variables that Affect Outcomes of Anesthesia for Surgery Patients”) and the analytical plan was published on clinicaltrials.gov before data collection and analysis (NCT02057653). Patient consent was waived as this was a quality improvement initiative. Since the study was initiated as a quality improvement project, it is reported following the Standards for Quality Improvement Reporting Excellence (SQUIRE guidelines) [16, 17] and is presented as a historical-prospective, comparative effectiveness format following the GRACE (Good Research for Comparative Effectiveness) initiative principles and checklist [18, 19] (Additional file 1).

From 1 June 2011 to 15 September 2013, all consecutive patients undergoing open colectomy (ICD-9 codes 45.71, 45.72, 45.73, 45.74, 45.75, 45.76, 45.79, 45.8, 45.82, 45.83, and 48.5), pancreatectomy (ICD-9 codes 52.5, 52.6 and 52.7), pelvic surgery with cancer debulking (ICD-9 codes 65.6, 66.5, 68.4, 68.59, 68.6, 68.7, and 68.9), and liver resection (ICD-9 codes 50.22 and 50.3) and equipped with an arterial line were considered the target of this study. Anesthesia providers in the Department of Anesthesiology and Perioperative Care at the University of California Irvine (47 attending anesthesiologists, 33 residents, and 18 certified nurse anesthetists) participated in this study. Patients less than 18 years old, pregnant women, emergency surgery, and patients admitted to the hospital more than 24 hours before surgery were excluded from this project.

### Designing the intervention

From 1 March 2012 to 1 June 2012, a group of five anesthesia team leaders and two surgeons was created to discuss issues related to change management. This group worked closely with the Chair (ZK) and the Vice Chair for Quality and Patient Safety (SV) in the Department of Anesthesiology and Perioperative Care and with Nursing, the IT Department, Intensive Care Unit physicians, and Anesthesia Technicians. The group met on a bi-weekly basis to design the protocols of care. The team leaders designed the fluid management protocols as well as the hemodynamic optimization protocol. The intervention consisted of a series of protocols for fluid administration and hemodynamic management during high-risk abdominal and pelvic surgeries. The use of an arterial line was decided on a case-by-case basis by the anesthesiologist who was providing the clinical care and was not affected by the study described in this report.

**Fig. 1:**
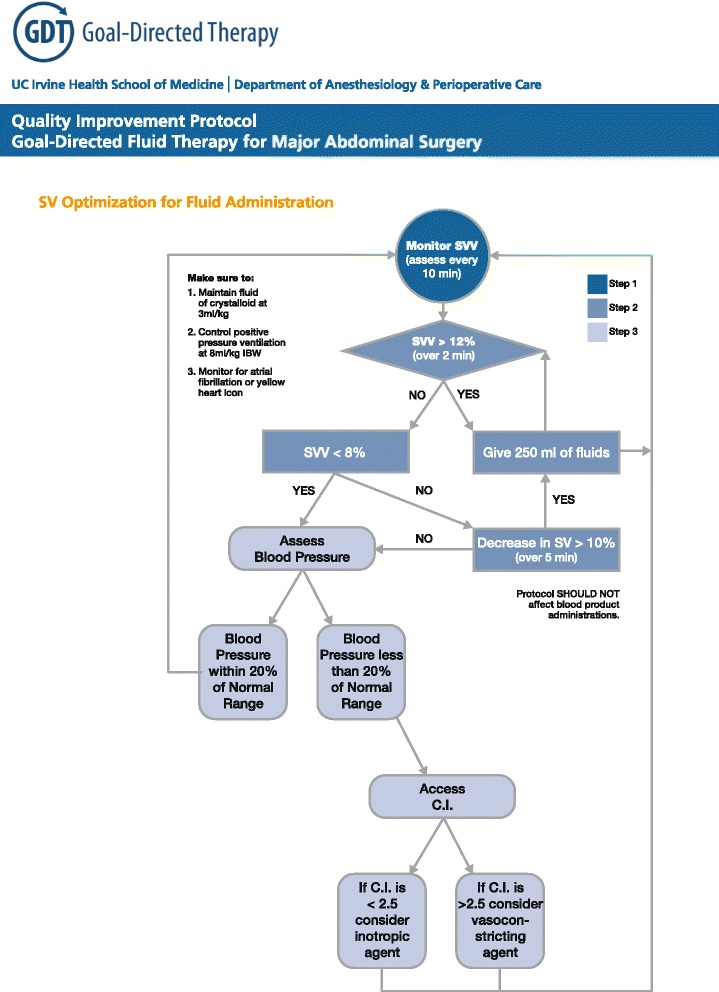
Perioperarive goal-directed algorithm. *C.I.* cardiac index, *IBW* ideal body weight, *SV* stroke volume, *SVV* stroke volume variation

The fluid management protocol consisted of standardized baseline crystalloid administration of 3 ml/kg/hour and any additional boluses based on hemodynamic monitoring endpoints (EV 1000, Edwards Lifesciences, Irvine, CA, USA). An infusion pump was used to deliver the fluids (Fig. 1). For the first 2 weeks of implementation (1 June 2012 to 15 June 2012), hemodynamic protocol was based on stroke volume optimization alone (additional 250 ml boluses of fluid for any >10 % decrease in stroke volume following the NICE protocol as recommended by the National Health Service in the United Kingdom [10]). However, the feedback from anesthesiology providers was that this protocol was forcing them to administer more fluids than they would feel comfortable administering and the team leaders decided to include stroke volume variation (SVV) as the trigger for fluid administration (Fig. 1) in order to increase the buy-in from clinicians. This protocol was not adapted to patient severity or to the type of surgery (open or laparoscopic) as we believed clinicians would modify their approach on their own based on the education provided.

### Implementing the intervention

#### Training period

From 19 May 2012 to 31 May 2012 all anesthesiology providers were asked to take an online test to evaluate the knowledge related to fluid management and hemodynamic optimization (Additional file 2). We achieved 100 % participation in this online test. On 1 June 2012 we published on the intranet of the Department of Anesthesiology and Perioperative Care a teaching website dedicated to PGDT and fluid management (information available here: gdt.anesthesiology.uci.edu (last access 20 August 2014)). This website included modules focusing on physiological concepts and clinical application tools related to PGDT. The clinical protocols for fluid management and PGDT are also accessible on the website and are accessible in all operating rooms in our organization. In addition, the week of the initiation of the training period, we organized the 2nd Goal Directed Therapy Symposium that consisted of a 2-day course on basic hemodynamic concepts and practical PGDT education (information available in [20] (last access 20 October 2014)). Finally, in June 2012 a joint Grand Rounds of the Departments of Anesthesiology and Perioperative Care and Surgery focusing on the initiative and its implementation was presented.

During this training period, once a patient appropriate for the study was identified, we ensured that the anesthesia providers applied the PGDT protocols as indicated. During this time period, team leaders of this initiative were available 7 days a week to help anesthesia to implement the protocol. During the surgical procedures, implementation of PGDT was tracked using a pop-up window appearing in the Anesthesia Information Management System (AIMS; Surgical Information Systems, Atlanta, GA, USA) during the anesthesia time-out before induction and requesting anesthesiologists to apply PGDT during the case. At the end of the surgery, before closing the AIMS record, another pop-up window would ask the anesthesiologist if the PGDT protocol was applied during the case. At the end of the training period (from 1 September 2012 to 15 September 2012) all anesthesiology providers took a post-training test to assess the change in knowledge that occurred during the training phase.

#### Launching period

On 15 September 2012 the official program was officially launched and clinicians were expected to apply the protocol for all high-risk abdominal and pelvic surgery patients equipped with an arterial line.

### Outcome measurement

To study the impact of the intervention on postoperative outcome, we compared the pre-implementation period (1 June 2011 to 31 May 2012) to the post-implementation period (1 June 2012 to 15 September 2013). In the pre- and post-implementation periods, outcome data were collected using our AIMS system, electronic medical records (EMR; Sunrise Clinical Manager, Allscripts, Chicago, IL, USA) (manual collection), and the National Surgical Quality Improvement Program (NSQIP) database available at our institution. In order to guarantee that data acquisition was the same in the two time periods, data collection variables were decided based on GRACE and SQUIRE guidelines [16, 17] (clinical data was recorded retrospectively in both time periods even though the PGDT program was implemented prospectively). Also, NSQIP data was collected on a daily basis for all colorectal, hepatobiliary, pancreatic, and pelvic surgeries.

#### Process measures

Adherence to the PGDT protocol application was defined as: 1) use of a pump for baseline crystalloid administration at 3 ml/kg/hour *and* 2) use of the cardiac output monitor *and* 3) documentation of PGDT in the AIMS record. We also recorded total intraoperative volumes of fluids administered, postoperative fluid balance and average tidal volume and positive end-expiratory pressure. Intraoperative volumes of fluids are reported in ml/kg/hour (total fluid administration defined as crystalloid + colloid, total crystalloid administration, total colloid administration). Postoperative fluid balance was defined as total intraoperative fluid administered – (estimated blood loss + intraoperative urine volume) and was expressed in ml [21].

#### Outcome measures

The primary outcome measure was LOS in the hospital after surgery defined as the number of nights spent in the hospital after the day of surgery. Secondary outcome measures included: intraoperative packed red blood cell transfusion, number of packed red blood cells transfused per patients transfused, average lowest intraoperative hemoglobin value, average intraoperative mean arterial pressure, central venous pressure, cardiac output and SVV, incidence of unplanned mechanical ventilation for more than 6 hours after surgery, incidence of postoperative complications, LOS in the intensive care unit (defined as the number of nights spent in the intensive care unit after the day of surgery), readmission to the hospital within 30 days after hospital discharge, and 30-day postoperative mortality (Table 1).

**Table 1:** Outcome measures collected for the analysis and their sources

Data	Acronym	Definition	Source
*Primary outcome*			
Post operative length of stay in the hospital	LOS	Number of nights in the hospital after surgery	Electronic Medical Record
*Secondary outcome*			
Intraoperative blood transfusion	Blood		AIMS
Postoperative mechanical ventilation >6 hours	Postop vent		Electronic Medical Record
Length of stay in the intensive care unit	LOS ICU	Number of nights in the intensive care unit after surgery	Electronic Medical Record
NSQIP complication	NSQIP comp	Incidence of at least one NSQIP complication from the list presented below	NSQIP database
Acute kidney injury	AKI	NSQIP definition	NSQIP database
Deep vein thrombosis	DVT	NSQIP definition	NSQIP database
Illeus	Ills		Electronic Medical Record
Myocardial infarction	MI	NSQIP definition	NSQIP database
Pneumonia	Pnmn	NSQIP definition	NSQIP database
Pulmonary embolism	PE	NSQIP definition	NSQIP database
Sepsis	Spss	NSQIP definition	NSQIP database
Stroke	Stk	NSQIP definition	NSQIP database
Surgical site infection	SSI	NSQIP definition	NSQIP database
Urinary tract infection	UTI	NSQIP definition	NSQIP database
30-day readmission	30 days readmission	Hospital readmission within 30 days after surgery	Electronic Medical Record

*AIMS* Anesthesia Information Management System, *NSQIP* National Surgical Quality Improvement Program

### Analysis

The primary goal of this study was to assess the impact of the protocol implementation on LOS in the hospital. To adjust for potential confounding variables for LOS, in addition to the intervention variable, we also included the following variables as covariates in our multiple linear regression analysis: surgery type, surgery duration in hours, American Society of Anesthesiologists score, estimated blood loss, body mass index, age, and laparoscopic or open procedure. Because LOS, surgery duration, estimated blood loss, and body mass index are skewed, we applied log-transformation to them. The *p*-value was calculated using the Wald test.

The analysis of other continuous outcomes is similar to that of LOS in the hospital. Log-transformation was applied when necessary. For outcomes that had a minimum value zero, we added 1.0 to all values before taking the log. For binary variables (intraoperative packed red blood cell transfusion, unplanned mechanical ventilation, postoperative complications, readmission to the hospital within 30 days after hospital discharge, and 30-day postoperative mortality) with two possible outcomes, “yes” or “no”, the Wald test from logistic regression was used to examine the impact of the implementation. To account for potential confounders for binary variables, we also used surgery type, surgery duration in hours, ASA score, estimated blood loss, body mass index, age, and laparoscopic or open procedure as covariates in our binomial logistic regression analysis.

All data are presented as mean ± SD, median (25th percentile to 75th percentile), or count (percentage) as appropriate. Using the parameters estimated from the multiple linear regression, the power of detecting association between LOS and the implementation of the protocol was greater than 90 %. All comparisons were made at a significance level of 0.05, and all analyses were performed with R [22] and SPSS 21.0.0.0 (IBM, Armonk, NY, USA).

## Results

### Nature of setting and improvement intervention

In the pre-implementation period, 128 subjects were included from 1 June 2011 to 1 June 2012. In the post-implementation period, 202 patients were included (42 during the training period from 1 June 2012 to 15 September 2012, and 160 post-training from 16 September 2012 to 15 September 2013). Demographics of patients included in the pre-implementation and post-implementation periods are presented in Table 2. A total of five surgeons were involved in the care of these patients. Table 2 shows the distribution of the main intraoperative procedures in the pre-implementation and post-implementation periods. All anesthesia providers involved in this study took the pre-implementation test and the post-implementation test. The average score before training was 62 ± 22 % compared to 78 ± 17 % at the end of the training period (*p* < 0.0001).

**Table 2:** Demographic data in the pre- and post-implementation periods

	Pre-implementation (*n* = 128)	Post-implementation (*n* = 203)	*p* value
Surgery			
Colorectal	27 (21 %)	38 (19 %)	0.72
Gynecology	19 (15 %)	22 (11 %)	0.37
Liver resection	20 (16 %)	48 (24 %)	0.1
Pancreatectomy	62 (48 %)	94 (46 %)	0.82
ASA			
II	14 (11 %)	17 (8 %)	0.75
III–IV	114 (89 %)	184 (91 %)	0.57
V	0 (0 %)	1 (<1 %)	0.68
Age (years)	66 ± 14	63 ± 14	0.05
Height (cm)	167 ± 10	167 ± 11	0.6
Weight (kg)	75 ± 19	76 ± 17	0.61
BMI (kg/m2)	3.3 ± 0.2	3.3 ± 0.2	0.35
Duration of surgery (hours)	7.0 ± 2.2	7.8 ± 2.8	0.005
Estimated blood loss (log)	5.7 ± 1.1	5.5 ± 1.2	0.08
Procedure			
Laparoscopic	42 (33 %)	79 (39 %)	0.3
Open	86 (67 %)	123 (61 %)	0.3

Two-sample *t*-test for continuous variables and Pearson’s chi-squared test for binary/categorical variables. *American Society of Anesthesiologists*, *BMI* Body Mass Index

### Changes in processes of care and patient outcomes associated with the intervention

#### Process of care

The frequency of usage of PGDT, as assessed by the criteria listed on page 9 was 7 % in the pre-implementation period and 61 % in the post-implementation period (*p* < 0.0001). Average total volume of fluids administered during a case decreased significantly from the pre-implementation period to the post-implementation period (9.9 (7.1–13.0) ml/kg/hour versus 6.6 (4.7–9.5) ml/kg/hour, *p* < 0.0001). This decrease was mainly related to a decrease in crystalloid administration in the pre-implementation period compared to the post-implementation period (7.5 (5.2–10.0) ml/kg/hour versus 4.9 (3.4–7.1) ml/kg/hour, *p* < 0.0001). The fluid balance (defined as total intraoperative fluid administered – (estimated blood loss + intraoperative urine volume)) was also significantly different between in the pre-implementation period and the post-implementation period (3,238 (2,459–4,412) ml versus 2,418 (1,671–3,636) ml, *p* = 0.003).

#### Primary outcome

**Fig. 2:**
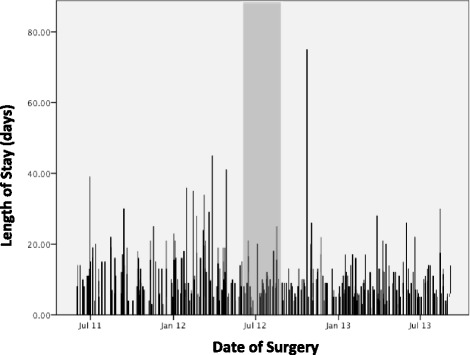
Time series analysis showing length of stay in the hospital (in days) for each patient over the total study period including pre-implementation period (*left side of the gray area*), training period (*gray area*), and post-implementation period (*right side of the gray area*)

LOS in the hospital decreased from the pre-implementation period to the post-implementation period (10 (6–16) days versus 7 (5 – 11) days, *p* = 0.0001) (Table 3). Based on the multiple linear regression analysis of LOS on a log scale, the estimated coefficient for intervention was 0.203 (SE = 0.054, *p* = 0.0002). This indicates that, with the other conditions being held the same, introducing intervention reduces LOS by 18 % (95 % confidence interval 9–27 %). The sequence chart showing LOS in the hospital for each day a patient was included from 1 June 2012 to 15 September 2013 is shown in Fig. 2.

**Table 3:** Outcome metrics in the pre- and post-implementation periods

	Pre-implementation (*n* = 128)	Post-implementation (*n* = 203)	*p* value	Odds ratio (95 % CI)
Primary outcome				
LOS in the hospital (nights)	10 (6–16)	7 (5–11)	NA	NA
LOS in the hospital (log transformed)	2.31 ± 0.62	2.03 ± 0.57	0.0002	NA
Secondary outcomes				
LOS in the ICU (nights)	1 (1–3)	1 (0–2)	NA	NA
LOS in the ICU (log 1+ LOS ICU)	0.97 ± 0.98	0.72 ± 0.68	0.02	NA
PRBC transfusion (n (%))	56 (43.8)	65 (32.2)	0.12	0.65 (0.38–1.12)
Units of PRBC transfused per patients transfused (n)	2 (1–4)	2 (1–3)	0.34	NA
Extubation within 6 hours after surgery (n (%))	102 (79.7)	174 (86.1)	0.21	1.66 (0.76–3.63)
NSQIP complication (n (%))	51 (39.8)	50 (24.8)	0.03	0.51 (0.31–0.93)
Type of complication (n (%))				
AKI	2 (1.6)	2 (1.0)	NA	NA
Delirium	0 (0.0)	1 (0.5)	NA	NA
DVT	4 (3.1)	3 (1.5)	NA	NA
Illeus	3 (2.3)	11 (5.4)	NA	NA
Myocardial infarction	0 (0.0)	2 (1.0)	NA	NA
Stroke	4 (3.1)	2 (1.0)	NA	NA
Pneumonia	11 (8.6)	7 (3.5)	0.35	0.60 (0.21–1.74)
Sepsis	0 (0.0)	1 (0.5)	NA	NA
SSI	21 (16.4)	17 (8.4)	0.03	0.44 (0.21–0.93)
UTI	6 (4.7)	5 (2.5)	NA	NA
30-day readmission (n (%))	35 (27.3)	38 (18.8)	0.14	0.65 (0.37–1.15)
30-day mortality (n (%))	1 (0.8)	2 (1.0)	NA	NA

*AKI* acute kidney injury, *CI*, confidence interval, *DVT* deep vein thrombosis, *ICU* intensive care unit, *LOS* length of stay, *NA* not applicable (or events were too rare for analysis), *NSQIP* National Surgical Quality Improvement Program, *PRBC* packed red blood cells, *SSI* surgical site infection, *UTI* urinary tract infection

#### Secondary outcomes

Secondary outcome results are shown in Table 3. The estimated coefficient for intervention from the multiple linear regression of log-LOS in the ICU was 0.17 (SE = 0.07, *p* = 0.02), indicating that introducing intervention shortened LOS in the ICU by 16 % (95 % confidence interval 3–27 %). The incidence of NSQIP complications was 40 % in the pre-implementation period compared to 25 % in the post-implementation period with an estimated odds ratio from multiple logistic regression of 0.51 (95 % confidence interval 0.31–0.93, *p* = 0.03); that is, the intervention decreased the odds of a postoperative complication by 49 %. Specifically, we observed a decrease in the incidence of surgical site infections (16.4 % compared to 8.4 %; *p*-value adjusted for covariates = 0.03).

### Comparison between patients in whom PGDT was reported and patients in whom PGDT was not reported in the post-implementation period

In the post-implementation period, PGDT was fully reported in 124 patients (61 %) (based on the adherence criteria detailed in the Methods section). When analysis was limited to the post-implementation period, the total volume of fluids administered during the study was 7.9 (6.0–11.4) ml/kg/hour in patients in whom PGDT was reported and 5.8 (4.4–8.1) ml/kg/hour in patients in whom PGDT was not reported (*p* < 0.0001). Crystalloid administration was 6.4 (4.5–9.5) ml/kg/hour in patients in whom PGDT was reported compared to 4.1 (2.7–5.8) ml/kg/hour in patients in whom PGDT was not reported (*p* < 0.0001). The fluid balance was not significantly different between the two groups (PGDT or no PGDT; 2,390 (1,685–4,100) ml in patients in whom PGDT was reported compared to 2,423 (1,653–3,525) ml in patients in whom PGDT was not reported; *p* < 0.002). LOS in the hospital in patients in whom PGDT was not fully reported was 8 (6–11) days compared to 8 (5–11) days in patients in whom PGDT was fully reported (*p* = 0.21).

## Discussion

Under the conditions of this quality improvement project we found that implementation of standardized PGDT strategies reduced LOS and the incidence of postoperative complications in patients undergoing high-risk abdominal and pelvic surgeries. This finding confirms what has been suggested in previously published randomized controlled trials in a “real life” setting. Indeed, several previous studies have suggested that the implementation of PGDT strategies for patients undergoing high-risk surgery has the ability to improve postoperative outcome [1, 3, 5, 6, 8]. Indeed PGDT is currently considered by experts as a cornerstone of the Enhanced Recovery After Surgery (ERAS) programs [23], and is recommended by several professional societies [9–12]. However, the application of this concept at the bedside has been slow and few clinicians apply it on a daily basis for their high-risk surgery patients [15]. As such, the purpose of this study was to test implementation within the context of ongoing routine clinical care rather than a trial.

Studies published in the past used different algorithms to drive the PGDT algorithm. Some used a stroke volume only based algorithm [3], while some used additional parameters such as SVV [24], corrected flow time [5], or central venous pressure. These algorithms may be different, but they all target the same kind of goal of bringing the heart function to the plateau of the Frank Starling curve, where any additional increase in preload would only create tissue edema and would not increase stroke volume/cardiac output anymore. A stroke volume only based algorithm would require additional fluid boluses to detect when the heart becomes preload independent (when stroke volume does not increase following an increase in preload) while an algorithm including a dynamic parameter of fluid responsiveness (such as SVV or PPV (pulse pressure variations)) would supposedly prevent any fluid administration to a preload-independent heart. In our experience, while we initially planned on implementing a stroke volume only based algorithm, we found that the acceptance of this approach was very low among the anesthesia providers. The introduction of SVV in the algorithm helped the implementation of this initiative. The main reason may be that during abdominal surgeries, a “zero balance” fluid administration is preferred to a liberal one (some studies even suggest that stroke volume only based algorithms may actually harm patients undergoing major abdominal surgery [25, 26]). Another approach, based on stroke volume alone, may be better accepted during other surgeries such as orthopedic surgery, for example. The drawback of using SVV/PPV is that it requires a tidal volume >7 ml/kg [27]. However, this would still remain a low tidal volume ventilation (6 to 8 ml/kg) and would qualify as protective lung ventilation strategies [28, 29]. The main advantage of a PPV-driven protocol is that this could be done free of charge using any monitor displaying an arterial pressure waveform and could be used in institutions that do not have a cardiac output/stroke volume monitor [30].

Recently, the implementation of PGDT strategies has been recommended as part of the ERAS programs [23]. The idea of this program is to put together a bundle of interventions that would each contribute to improved outcome to a certain extent. In this report, the full ERAS bundle was not applied consistently to all patients so the results from our initiative have to be interpreted accordingly. As a matter of fact, it is possible that the impact of the PGDT intervention would not be observed in a system where all the other items of the ERAS package are already implemented. Considering the cost of the hemodynamic monitoring system, studies examining the impact of PGDT implementation in an otherwise comprehensive ERAS program are warranted to evaluate the cost effectiveness of this approach. In addition, our protocol did not require specific changes based on patient condition (aerobically fit patients [25]) or type of surgery. It may be that adopting different protocols to different patient populations or type of surgery may further improve outcome. This would need to be evaluated.

Another potential improvement for the future will be related to techniques/strategies aimed at increasing the compliance to the protocol. In our study, we achieved 62 % compliance to the protocol. This is close to what has been reported in systems which have national recommendations such as the United Kingdom but lower than our experience with PGDT in a Perioperative Surgical Home model for joint replacement surgery as we recently described [14]. The Perioperative Surgical Home model of care may achieve much higher compliance to this protocol by applying human engineering tools such as LEAN or Six Sigma [13]. Another way to increase compliance/adherence to PGDT protocols would be the use of closed-loop or semi closed-loop systems that would apply the protocol in an automatic or semi-automatic way [31]. It is not possible to definitively make conclusions on patients who received the full PGDT protocol compared to those who did not because the definition of compliance to the protocol was not tracked the way it would have been in a research study with research coordinators and researchers (and hemodynamic data such as cardiac output and SVV was not continuously and consistently recorded for all patients). It may actually be that in the post-implementation period, patients who did not fall under our definition of PGDT – 1) use of a pump for baseline crystalloid administration at 3 ml/kg/hour *and* 2) use of the cardiac output monitor *and* 3) documentation of PGDT in the AIMS record – actually received PGDT while some who did fall under the definition actually did not receive it. One of the reasons for this observation may be a learning contamination bias which would explain that patients for whom PGDT was not reported in the post-implementation phase were actually treated differently from those in the pre-implementation period because clinicians were getting used to PGDT protocols. This hypothesis has been discussed recently in letters related to the ARISE trial [32, 33]. Further studies evaluating strategies to increase compliance, such as incorporation in a Perioperative Surgical Home model [13, 14] are thus needed. It may also mean, as suggested by previously published studies, that a crystalloid restriction strategy alone may be able to improve outcome in this population.

Finally, we did not ask clinicians in our department to use a specific type of fluid to conduct PGDT. Considering the controversy surrounding crystalloids versus colloids at the time of the study we did not want to recommend anything. However, one has to keep in mind that during the period of the study the FDA released a black box warning regarding hydroxyethyl starch and we decided to remove this drug from our operating rooms. Of note, we did not find any difference in the incidence of postoperative acute kidney injury (Table 3).

### Limitations

Quality improvement studies are inherently limited because they are not randomized or controlled and they reduce the ability to make a causal connection between the intervention and the change in outcome. However, when implementing a complex process (such as PGDT) it is practically impossible to randomize and to control the implementation of the process under test. For this reason, the historical-prospective approach methodology is one of the methodologies available for testing the implementation of a quality improvement program in the setting of comparative effectiveness research. This approach has been used in several studies, including some with significant impact on healthcare and perioperative medicine [34]. In addition, we have used multivariate regression analysis to strengthen our conclusion and increase the repeatability of our results. However, one has to keep in mind that the changes in outcome observed in our experience may have been related to other sources, especially considering the recent pressure for decreasing cost, improving care, and increasing overall quality in all healthcare systems in the US [35]. Since the goals of the program were to detect meaningful changes and there were implications for dissemination far beyond a single institution, our project is more than a simple quality improvement project. We implemented a system redesign with testing at a local institution to define what elements can be standardized and exported. This is similar in some ways to an efficacy trial and this would not have been achievable in a standard randomized controlled trial.

Another limitation may be in the reporting of postoperative outcomes that could differ between the historical and the prospective groups. However, we collected data retrospectively and used the same data collection methods in the historical and in the prospective group. In addition, the primary outcome is the LOS in the hospital (which is an objective variable), and we used the NSQIP database to standardize the way postoperative complications were reported.

## Conclusion

In conclusion, in our experience, the implementation of PGDT strategies leads to a change in fluid administration associated with improved postoperative outcome and decreased LOS in the hospital. This quality improvement/comparative effectiveness study confirms what has been suggested by previously published randomized controlled trials.

## Key messages

- The implementation of PGDT strategies for high-risk surgery patients leads to a change in fluid administration associated with improved postoperative outcome and decreased LOS in the hospital.
- This finding confirms what has been suggested in previously published randomized controlled trials in a “real life” setting.
- Another potential improvement for the future will be related to techniques/strategies aimed at increasing the compliance to the protocol. In our study, we achieved 62 % compliance to the protocol.

## Additional files

Additional file 1:
**Grace Checklist.**
Additional file 2:
**Pre and post online quiz.** Questionnaire used to assess knowledge before and after training.

## Abbreviations

AIMS: Anesthesia Information Management System
ASA: American Society of Anesthesiologists
ERAS: Enhanced Recovery After Surgery
LOS: Length of stay
NSQIP: National Surgical Quality Improvement Program
PGDT: Perioperative goal-directed therapy
PPV: Pulse pressure variations
SVV: Stroke volume variation
