# Supplementary material for: Perioperative goal-directed therapy and postoperative outcomes in patients undergoing high-risk abdominal surgery: a historical-prospective, comparative effectiveness study
Source: Crit Care. 2015 Jun 19;19(1):261. doi: 10.1186/s13054-015-0945-2 (PMC4512146; doi:10.1186/s13054-015-0945-2)
Supplement: Additional file 1: — Grace Checklist. [file 13054_2015_945_MOESM1_ESM.pdf]

grace PRINCIPLES

A Validated Checklist

for Evaluating the Quality of Observational Cohort Studies for Decision-Making Support

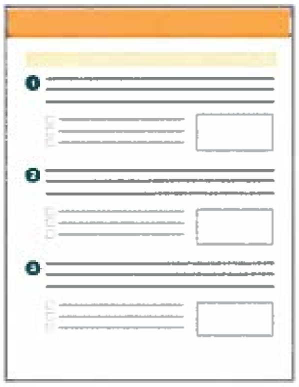

GRACE: Good ReseArch for Comparative Effectiveness

The GRACE Checklist is designed for the assessment of observational studies of comparative effectiveness in terms of their quality and usefulness for decision-making. The checklist was developed from a review of the literature with guidance from recognized experts in this field. The content includes questions about data and methods. Validation activities have documented the usefulness of all 11 questions in this checklist. Approaches to scoring are under consideration.

The GRACE Initiative has been spearheaded by Quintiles Real-World & Late Phase Research in collaboration with the National Pharmaceutical Council. GRACE contributors represent perspectives from academic, government, and private sectors in the US, Europe, Asia and Africa. More information, including a list of contributors and collaborators can be found at www.graceprinciples.org. More information is available in the American Journal of Managed Care 2010; 16(6): 467-471 (Dreyer NA, Schneeweiss S, McNeil B et al.) and the Journal of Managed Care & Specialty Pharmacy 2014; 20(3):301-8.

To join the GRACE Initiative or for more information, please contact us at coordinator@graceprinciples.org. Feedback welcomed.

Nancy A. Dreyer
Leader, GRACE Initiative

ALWAYS CONSIDER: *Is this adequate for the study purpose?*

Data

D1 **Were treatment and/or important details of treatment exposure adequately recorded for the study purpose in the data source(s)?** *Note: not all details of treatment are required for all research questions.*

- [x] **Yes**—reasonably necessary information to determine treatment or intervention was adequately recorded for study purposes (e.g., for drugs, sufficient detail on dose, days supplied, route or other data important. For vaccines, consider the importance of batch, dose, route and site of administration, etc. For devices, consider type of device, placement, surgical procedure used, serial number, etc.).
- [ ] **No**—data source clearly deficient ***or* not enough information in article**

Comments: Described in Method section.

D2 **Were the primary outcomes adequately recorded for the study purpose (e.g., available in sufficient detail through data source(s))?**

- [x] **Yes**—information to ascertain outcomes were adequately recorded in the data source (e.g., if clinical outcomes were ascertained using ICD-9 diagnosis code(s) in an administrative database, the level of sensitivity and specificity captured by the code(s) was sufficient for assessing the outcome of interest.)
- [ ] **No**—data source clearly deficient (e.g., the code(s) captured a range of conditions that was too broad or narrow, and supplementary information such as that from medical charts was not available), ***or* not enough information in article**

Comments:

D3 **Was the primary *clinical* outcome(s) measured objectively rather than subject to clinical judgment (e.g., opinion about whether the patient's condition has improved)?**

- [x] **Yes**—clinical outcomes were measured objectively (e.g., hospitalization, mortality)
- [ ] **N/A**—primary outcome not clinical (e.g., PROs)
- [ ] **No**—e.g., clinical opinion about whether patient's condition improved, ***or* not enough information in article**

Comments:

ALWAYS CONSIDER: *Is this adequate for the study purpose?*

D4 **Were primary outcomes validated, adjudicated, or otherwise known to be valid in a similar population?**

- [x] **Yes**—outcomes were validated, adjudicated, or based on medical chart abstractions with clear definitions, e.g. a validated instrument was used to assess patient-reported outcomes (e.g., SF-12 Health Survey); a clinical diagnosis via ICD-9 code was used, with formal medical record adjudication by committee to confirm diagnosis or other procedures to achieve reasonable sensitivity and specificity; billing data were used to assess health resource utilization, etc.
- [ ] **No, *or* not enough information in article**

Comments:

D5 **Was the primary outcome(s) measured or identified in an equivalent manner between the treatment/ intervention group and the comparison group(s)?**

- [x] **Yes**
- [ ] **No, *or* not enough information in article**

Comments:

D6 **Were important covariates that may be known confounders or effect modifiers available and recorded?** *Important covariates depend on the treatment and/or outcome of interest, (e.g., body mass index should be available and recorded for studies of diabetes; race should be available and recorded for studies of hypertension and glaucoma).*

- [x] **Yes**—most if not all important known confounders and effect modifiers available and recorded, e.g., measures of medication dose and duration.
- [ ] **No**—at least one important known confounder or effect modifier not available and recorded (as noted by authors or as determined by user's clinical knowledge), ***or* not enough information in article**

Comments:

Methods

M1 **Was the study (or analysis) population restricted to new initiators of treatment or those starting a new course of treatment?** *Efforts to include only new initiators may include restricting the cohort to those who had a washout period (specified period of medication nonuse) prior to the beginning of study follow-up.*

- [x] **Yes**—only new initiators of the treatment of interest were included in the cohort, or for surgical procedures and devices, only patients who never had the treatment before the start of study follow-up were included.
- [ ] **No, *or* not enough information in article**

Comments:

ALWAYS CONSIDER: *Is this adequate for the study purpose?*

M2 **If one or more comparison groups were used, were they concurrent comparators? If not, did the authors justify the use of historical comparisons group(s)?**

- [x] **Yes**—data were collected during the same time period as the treatment group ("concurrent") or historical comparators were used with reasonable justification, e.g., when it is impossible for researchers to identify current users of older treatments or when a concurrent comparison group is not valid—(i.e., uptake of new product is so rapid that concurrent comparators differ greatly on factors related to the outcome)
- [ ] **No**—historical comparators used without being scientifically justifiable, ***or*** **not enough information in article**

Comments:

M3 **Were important covariates, confounding and effect modifying variables taken into account in the design and/or analysis?** *Appropriate methods to take these variables into account may include: restriction, stratification, interaction terms, multivariate analysis, propensity score matching, instrumental variables or other approaches.*

- [x] **Yes**—most if not all important covariates that would be likely to change the effect estimate substantially were accounted for, e.g., measures of medication dose and duration.
- [ ] **No**—some important covariates were available for analysis but not analyzed appropriately, ***or*** **at least one important covariate was not measured, *or* not enough information in article**

Comments:

M4 **Is the classification of exposed and unexposed person-time free of "immortal time bias"?** *Immortal time in epidemiology refers to a period of cohort follow-up time during which death (or an outcome that determines end of follow-up) cannot occur.*

- [x] **Yes**
- [ ] **No, *or* not enough information in article**

Comments:

M5 **Were any meaningful analyses conducted to test key assumptions on which primary results are based?** *E.g., were some analyses reported to evaluate the potential for a biased assessment of exposure or outcome, such as analyses where the impact of varying exposure and/or outcome definitions was tested to examine the impact on results.*

- [ ] **Yes**—and primary results *did not* substantially change
- [ ] **Yes**—and primary results changed substantially
- [x] **None reported, *or* not enough information in article**

Comments:

grace | Good ReseArch for Comparative Effectiveness
