# Supplementary material for: Perioperative goal-directed therapy and postoperative outcomes in patients undergoing high-risk abdominal surgery: a historical-prospective, comparative effectiveness study
Source: Crit Care. 2015 Jun 19;19(1):261. doi: 10.1186/s13054-015-0945-2 (PMC4512146; doi:10.1186/s13054-015-0945-2)
Supplement: Additional file 2: — Pre and post online quiz. Questionnaire used to assess knowledge before and after training. [file 13054_2015_945_MOESM2_ESM.pdf]

Pre-Online Module Quiz on GDT Hemodynamic Monitoring

***1. Please identify your position within the department**

- A. Resident
- B. CRNA
- C. Attending

***2. In the setting of a patient under controlled mechanical ventilation undergoing an open abdominal procedure, which of the following is the best predictor of fluid responsiveness?**

- 1. Heart Rate
- 2. Urine Output
- 3. Stroke Volume Variation
- 4. Central Venous Pressure
- 5. Pulmonary Wedge Pressure

***3. Which is the most effective primary intervention to improve CO in a patient who has low blood pressure and high stroke volume variation (SVV)?**

- a. A vasopressor will simultaneously get MAP to a target level and “shrink the tank” thereby reducing “relative hypovolemia” and improving SVV.
- b. A vasopressor because it’s more important to treat hypotension than high SVV.
- c. Fluids will decrease SVV and increase MAP, potentially preempting the need for vasopressors which could increase afterload and thereby decrease CO.
- d. Fluids or pressor as along as the MAP is improved to target level

***4. Aside from hypovolemia, what other conditions can also increase the variation in stroke volume (SVV):**

- a. Arrhythmia
- b. Cardiac tamponade
- c. Induction of anesthetics
- d. Prone positioning
- e. All of the above

***5. Giving an inotrope to a hypovolemic patient...**

- a. can be an effective way to improve CO when you require a dry surgical field.
- b. will increase CO by increasing the contractility of the heart, compensating for decreased preload.
- c. will increase CO by causing a big increase in HR.
- d. will have no effect because the increased contractility does not compensate for decreased preload.
- e. None of the above.

***6. A patient can lose up to ___% of their circulating blood volume before you see any changes in blood pressure.**

- a. 10
- b. 15
- c. 20
- d. 30

***7. SVV can be used to predict fluid responsiveness when all of the following have been met except?**

- A. Controlled positive pressure ventilation at 8ml/kg of ideal body weight
- B. Regular cardiac conduction (regular rhythm)
- C. Intact pulmonary pleura
- D. Patient has full muscle relaxation

***8. The definition of fluid responsiveness is a increase of which percentage in stroke volume with a 200ml fluid bolus?**

- a. 10
- b. 20
- c. 30
- d. 40

***9. Which is the most effective primary intervention to improve CO in a patient who has an elevated SVV and also a high SVR?**

- a. Fluids, because it will improve preload and CO which will in turn reduce SVR, potentially preempting the need for a vasodilator.
- b. Vasodilator, because the high SVR (afterload) is impeding cardiac output, and would also counteract the benefits to CO from improving preload with fluids.
- c. Fluids, and optionally an inotrope, because a vasodilator would make this patient more hypovolemic.
- d. Both fluids and vasodilator, to simultaneously lower SVV and SVR .
- e. The answer depends on the clinical objective and the clinician's school of thought on how to best optimize CO.

***10. Administering inotropes, vasodilators or vasopressors are unlikely to have any effect on SVV.**

- a. True
- b. False

***11. Prone positioning causes the following change to SVV:**

- a. Increase in SVV
- b. Decrease in SVV

***12. A passive leg raise {PLR) is a pseudo volume challenge of approximately how many cc/ml of fluid?**

- a. 100cc
- b. 200cc
- c. 300-400cc
- d. 1000cc

***13. ScvO2 is affected by what four factors**

- a. Height, Weight, SVR, CO
- b. CO, Hgb, O2, and O2 consumption
- c. SVR, CVP, CO, DO2
- d. None of the above

***14. How does PEEP affect preload**

- a. PEEP increases intrathoracic pressures which decreases preload via compression on the vena cava.
- b. PEEP decreases intrathoracic pressure and assists RV filling
- c. Regarding the left side of the heart only, PEEP increases LA filling
- d. Regarding the left side of the heart only, PEEP decreases LA filling
- e. A and C

***15. Regarding the arterial waveform, the upslope of the waveform corresponds to?**

- A. Systemic Vascular Resistance
- B. Ventricular Contractility
- C. Left Ventricular End Diastolic Pressure
- D. None of the Above

***16. Regarding the arterial waveform, the downslope of the waveform corresponds to?**

- a. Systemic Vascular Resistance
- b. Ventricular Contractility
- c. Left Ventricular End Diastolic Pressure
- d. None of the Above

***17. A 1mmHG change in CVP when the patient is at the initiation of the stressed preload volume can result in how much of ml/min change in cardiac output?**

- A. 100 ml/min
- B. 300 ml/min
- C. 500 ml/min
- D. 1000 ml/min

***18. If one has access to dynamic parameters (SVV & CO,) and the patient has a stroke volume variation of less 10% and is still hypotensive the next step to decide appropriate treatment would be?**

- A. Give a vasoactive agent
- B. Assess the Cardiac output
- C. Give a fluid bolus
- D. Give an inotropic agent

***19. What is the target tidal volume one aims for positive pressure ventilation to assure accurate utility of stroke volume variation of >10% to predict fluid responsiveness.**

- a. 4ml/kg
- b. 5ml/kg
- c. 8ml/kg
- d. 10ml/kg

***20. A increase in respiratory rate above a rate of 30 has been shown to cause what to the generation of variation in stroke volume.**

- A. SVV will increase
- B. SVV will Decrease

***21. One's position on the Frank Starling curve is dependent on all of the following except.**

- a. Ventricular Contractility
- b. Preload
- c. Afterload
- d. All of the above affect the Frank Starling curve
